# Community species diversity mediates the trade‐off between aboveground and belowground biomass for grasses and forbs in degraded alpine meadow, Tibetan Plateau

**DOI:** 10.1002/ece3.8048

**Published:** 2021-08-29

**Authors:** Tiancai Zhou, Jian Sun, Ning Zong, Ge Hou, Peili Shi

**Affiliations:** ^1^ Key Laboratory of Ecosystem Network Observation and Modelling Institute of Geographic Sciences and Natural Resources Research Chinese Academy of Sciences Beijing China; ^2^ College of Resources and Environment University of Chinese Academy of Sciences Beijing China; ^3^ State Key Laboratory of Tibetan Plateau Earth System, Resources and Environment (TPESRE) Institute of Tibetan Plateau Research Chinese Academy of Sciences Beijing China

**Keywords:** alpine meadow, biomass allocation, degradation, plant function groups, strategy

## Abstract

Although many empirical experiments have shown that increasing degradation results in lower aboveground biomass (AGB), our knowledge of the magnitude of belowground biomass (BGB) for individual plants is a prerequisite for accurately revealing the biomass trade‐off in degraded grasslands. Here, by linking the AGB and BGB of individual plants, species in the community, and soil properties, we explored the biomass partitioning patterns in different plant functional groups (grasses of *Stipa capillacea* and forbs of *Anaphalis xylorhiza*). Our results indicated that 81% and 60% of the biomass trade‐off variations could be explained by environmental factors affecting grasses and forbs, respectively. The change in community species diversity dominated the biomass trade‐off via either direct or indirect effects on soil properties and biomass. However, the community species diversity imparted divergent effects on the biomass trade‐off for grasses (scored at −0.72) and forbs (scored at 0.59). Our findings suggest that plant communities have evolved two contrasting strategies of biomass allocation patterns in degraded grasslands. These are the “conservative” strategy in grasses, in which plants with larger BGB trade‐off depends on gigantic roots for soil resources, and the “opportunistic” strategy in forbs, in which plants can adapt to degraded lands using high variation and optimal biomass allocation.

## Graphical abstract

Two contrasting strategies of biomass allocation patterns were detected in grasses and forbs. “Conservative” strategy in grasses, in which plants with belowground trade‐off depend on gigantic roots for soil resources. “Opportunistic” strategy in forbs, in which plants can adapt to degraded lands via high variation and optimal biomass allocation.

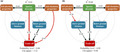

## 1 INTRODUCTION

The trade‐off between aboveground biomass (AGB) and belowground biomass (BGB) reflects the response and adaptation strategies to deal with environmental stress (Cheng & Niklas, 2007; Roa‐Fuentes et al., 2012), and many studies have been conducted to test whether biomass partitioning to AGB and BGB is isometric or allometric (Niklas, 2005; Sun et al., 2018). Specifically, an isometric biomass partitioning pattern has been detected in the forest (Yang & Luo, 2011) and grassland (Wang, 2017; Yang et al., 2010) ecosystems along climate gradients. However, on the Tibetan Plateau, several recent studies have demonstrated divergent biomass partitioning in grasslands due to grazing (Sun, Ma, et al., 2018) and degradation (Peng, Xue, You, et al., 2020).

**FIGURE 1:**
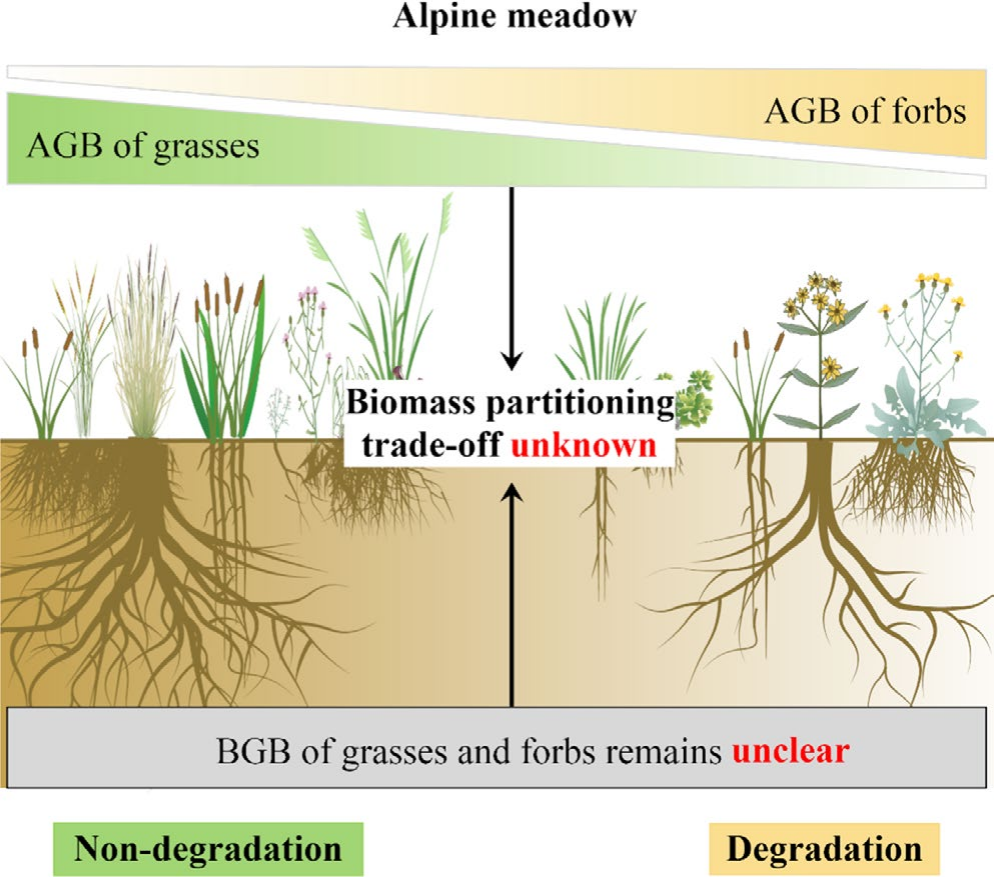
The diagram of the research gap in our study. An accurate revelation of the biomass trade‐off in degraded grasslands is based on our knowledge of the magnitude of aboveground biomass (AGB) and belowground biomass (BGB) for individual plants

Although these recent studies have enhanced our knowledge of the environment and plant trade‐off relationships (Roa‐Fuentes et al., 2012; Yang et al., 2010), very little is known about the BGB and the biomass partitioning of individual plants with different functions in degraded alpine meadow. Specifically, Sun, Ma, et al. (2018) and Peng, Xue, Li, et al. (2020) jointly gathered different plant roots using soil cores of the same depth in degraded grasslands. However, grasses tend to extend horizontally shallow roots in the surface soil (Liu, Zhang, Sun, Li, et al., 2020; Peng, Xue, You, et al., 2020), and forbs always produce deep axial roots to absorb soil nutrients after grassland degradation (Peng, Xue, Li, et al., 2020; Zhang et al., 2020). These plant root distribution changes may then trigger various biomass partitioning trade‐offs at different soil depths. Hence, our knowledge of the magnitude of AGB and BGB for individual plants is a prerequisite for accurately revealing the biomass trade‐off in grassland degradation (Figure 1).

Meanwhile, many studies have explored the mechanisms of biomass partitioning in response to environmental factors (Roa‐Fuentes et al., 2012; Xu et al., 2010). However, only 7% and 13% of the variations in the roots and shoots worldwide are explained by annual mean precipitation and temperature (Yang et al., 2010). This suggests that there might be other factors determining biomass partitioning, such as community species diversity (Mokany et al., 2006; Yang et al., 2010). Community species diversity plays a critical role in shaping the trade‐off through its effects on AGB (Cardinale et al., 2007; Fraser et al., 2015) and BGB (Rajaniemi et al., 2003). High species diversity generally generates high grasses’ AGB, but high forb's AGB is usually found in the subclimax community (Wu et al., 2019). Meanwhile, owing to a significant decrease in community species in alpine meadow, grasses tend to increase BGB to improve soil nutrient uptake in degraded alpine meadow (Peng, Xue, You, et al., 2020). In contrast, forbs always extend their leaf area to enhance carbon fixation and AGB (Liu, Zhang, Sun, Li, et al., 2020; Siebert & Dreber, 2019). Therefore, exploring how the species diversity directly or indirectly influences the biomass allocation trade‐offs via soil properties in different functional groups is of paramount importance to understand the degradation process of alpine meadow.

According to the self‐constraint‐balance growth hypothesis (Sun et al., 2018), plant biomass allocation patterns involve a trade‐off in their life histories (Weiner, 2004). Although plants may preferentially allocate more biomass to their roots to absorb the limited soil nutrients in degraded grasslands (Shipley & Meziane, 2002; Sun, Niu, et al., 2018), there might be different plant biomass allocation patterns in response to degradation in plant communities. Grasses develop gigantic root systems to belowground in healthy alpine meadow (Peng, Xue, You, et al., 2020; Wu et al., 2017); while forbs in degraded grassland always show a more robust competitive capacity for light in the aboveground (Wang et al., 2009). Therefore, a better understanding of biomass trade‐offs within different plant functions is beneficial for revealing the process and mechanism of degradation. Here, we conducted a field survey in degraded alpine meadow to measure the individual AGB and BGB of different plant functional groups (grasses and forbs). Together with soil properties, these data were applied to test our hypothesis, which states that there might be different mechanisms shaping the biomass trade‐off in grasses and forbs after grassland degradation. Specifically, we aimed to (a) compare the biomass partitioning patterns in different plant functional groups along degradation gradients and (b) highlight the underlying mechanisms of how factors affect the variation in biomass trade‐off.

## 2 MATERIALS AND METHODS

### 2.1 Study area

Our study area is located in an alpine meadow in Damxung County (91°05E, 30°29N, ~4,313 m above sea level). In 2013, the annual mean temperature and precipitation are 2.4°C and 447.3 mm, respectively. Moreover, precipitation occurs during the summer monsoon, with ~90% of the precipitation arriving between May and September. The monthly mean temperature changes significantly, with the coldest (−8.6°C) and the warmest (12°C) months being January and July, respectively.

### 2.2 Identification of degradation

Because of overgrazing and climate change in recent years, alpine meadow in Damxung has experienced severe degradation (Zong & Shi, 2019). Specifically, alpine meadow has been invaded by *Anaphalis xylorhiza* (Zong et al., 2018). Following the method described by Peng, Xue, You, et al. (2020) and Zhou et al. (2021), three typical degraded gradients (nondegraded grassland [ND], moderately degraded grassland [MD], and severely degraded grassland [SD]) in the alpine meadow were defined based on the key criteria of plant coverage and community species composition (Table 1). It was found that the vegetation in nondegraded grassland was dominated by grasses of *Stipa capillacea* (Figure S1a); forbs of *A. xylorhiza* were dominant in degraded grasslands (Figure S1c).

**TABLE 1:** Location, altitude, vegetation, soil physical properties, and soil chemical properties of the sampling sites

Level	Longitude	Latitude	Altitude	Dominant species	Values	COV (%)	SWC (%)	ST (°C)	SBD (g/cm^3^)	SC (Pa)	STC (g/kg)	STN (%)	STP (mg/kg)
Non‐degradation	91°04′	30°30′	4,413 m	*Stipa capillacea*, *Kobresia pygmea* and *Carex montis everestii*	Min	70.00	10.87	8.1	0.97	51.07	16.90	0.30	746.10
					Mean	82.00	13.47	9.2	0.99	56.31	17.66	0.32	766.63
					Max	88.00	16.67	10.0	1.02	59.72	18.19	0.33	795.40
					Std	5.31	2.45	0.8	0.02	3.76	0.55	0.01	20.97
Moderate degradation	91°04′	30°30′	4,413 m	*Stipa capillacea*, *Carex montis‐everestii*, *Anaphalis xylorhiza*, *Artemisia*	Min	51.00	10.46	9.6	1.03	27.29	14.37	0.29	600.60
					Mean	54.00	11.21	10.7	1.08	31.86	15.75	0.30	644.67
					Max	60.00	12.14	12.4	1.15	38.08	16.65	0.31	684.70
					Std	4.32	0.70	1.2	0.05	4.56	0.99	0.01	34.45
Severe degradation	91°04′	30°30′	4,414 m	*Anaphalis xylorhiza*, *Artemisia*, *Potentilla bifurca*	Min	18.00	9.27	13.1	1.15	16.95	12.09	0.27	592.30
					Mean	21.00	9.82	14.1	1.19	19.33	12.55	0.28	637.67
					Max	26.00	10.57	15.0	1.25	22.38	12.96	0.29	681.80
					Std	3.40	0.55	0.8	0.04	2.26	0.36	0.00	36.55

Note

Mean, Min, Max, and Std indicate the average, minimum, maximum, and standard deviation values, respectively.

Abbreviations: COV, coverage; SBD, soil bulk density; SC, soil compactness; ST, soil temperature; STC, soil total carbon; STN, soil total nitrogen; STP, soil total phosphorus; SWC, soil water content.

### 2.3 Data collection

Three quadrats (50 × 50 cm) were randomly selected within each degraded site to determine certain aspects of the community species (composition, height, coverage, and the number of species). Next, two dominant species, including grasses of *S. capillacea* and forbs of *A. xylorhiza*, were collected to obtain the individual aboveground and belowground parts of the plants in August 2019. Thus, in each site, nine samples of each of these two species that grow individually were collected by excavating. Meanwhile, in each site, the aboveground and belowground in the community were oven‐dried at 65°C for 48 hr and weighed to 0.01 g, as AGB and BGB in the laboratory (Sun et al., 2018).

In each quadrat, soil temperature was measured using portable thermocouple TR‐8D (Shunkeda) at a soil depth of 0–20 cm. Later, soil samples were excavated using an auger which yielded 5 cm‐diameter soil cores from the topsoil of 0–20 cm. Then, soil moisture was determined with the drying method at 65°C. And the soil samples were sieved through a 2‐mm mesh after being air‐dried (Zhou et al., 2020). Finally, a series of soil properties were measured, including properties of soil total phosphorus (STP), measured by the molybdate colorimetric test with the perchloric acid digestion method; soil total carbon (STC) and soil total nitrogen (STN) measured by MACRO cube elemental analyzer (Elementar Analysensysteme GmbH) (Zhou et al., 2020).

### 2.4 Data analysis

#### 2.4.1 Plant diversity and biomass

The plant diversity indexes of the *Shannon–Wiener Index*, *Simpson Index*, *Margalef Index*, and *Pielou Index* were calculated using standard methods (Wang et al., 2009). A one‐way ANOVA was used to determine the influence of grassland degradation on the biomass of grasses and forbs.

#### 2.4.2 Calculation of trade‐off between AGB and BGB

The root mean square error was employed to determine the trade‐off between AGB and BGB for the two groups (grasses and forbs) (Sun, Ma, et al., 2018). In each quadrat, the relative benefits (RB) of biomass aboveground or belowground in each group were calculated by:

$$\text{RB}=\frac{x_{i}‐x_{\min}}{x_{\max}‐x_{\min}}$$

where $x_{i}$, $x_{\min}$, and $x_{\max}$ are the observed, lowest, and highest values of either AGB or BGB. In addition to this, the root mean square error indicates the zero trade‐off objective (1:1 line), which denotes the magnitude of benefits in AGB or BGB. The direction (whether above the 1:1 line or below the 1:1 line) of the trade‐off was also determined (Peng, Xue, You, et al., 2020; Sun, Ma, et al., 2018).

#### 2.4.3 Relations between the trade‐off and environmental factors

There are strong intercorrelations among community species diversity indexes (*Shannon–Wiener Index*, *Simpson Index*, *Margalef Index,* and *Pielou Index*), soil physical properties (soil temperature, moisture, compactness, and bulk density), and soil chemical properties (STC, STN, and STP) (Figure S2). In light of this, principal component analysis (PCA) was first conducted in R (R Core Team, 2015) (with packages of “*FactoMineR*,” “*factoextra,*” and “*corrplot*”) to extract the dominating components for each group before correlation analysis (Zhou et al., 2020). The results of which showed that 87.7%, 81.4%, and 84.1% (Figure S3) of the variance in community species diversity, soil physical properties, and soil chemical properties were explained by the first component (PC1), respectively. Hence, PC1 was introduced as a new variable representing each group factor and was used in the correlation analysis.

For the grass and forb, the relationships between the trade‐off and environmental factors along degraded gradients were explored by R (using the “*PerformanceAnalytics*” package) and visualized using the “*circlize*” package.

#### 2.4.4 Structural equation modeling

Finally, to reveal the ecological effect of key factors (selected from correlation analysis) on the trade‐off for the grasses and forbs, structural equation modeling (SEM) was conducted by Amos software (17.0.2, Amos Development Corporation). SEM is a multivariate statistical model that includes factors, paths, and maximum likelihood analysis (Zhou et al., 2020). In testing the hypothetical model, SEM is efficient in identifying critical variables, the direct and indirect effects of variables on the object variable can be separated by SEM (Zhou et al., 2020).

## 3 RESULTS

### 3.1 Plant biomass of grasses and forbs

**FIGURE 2:**
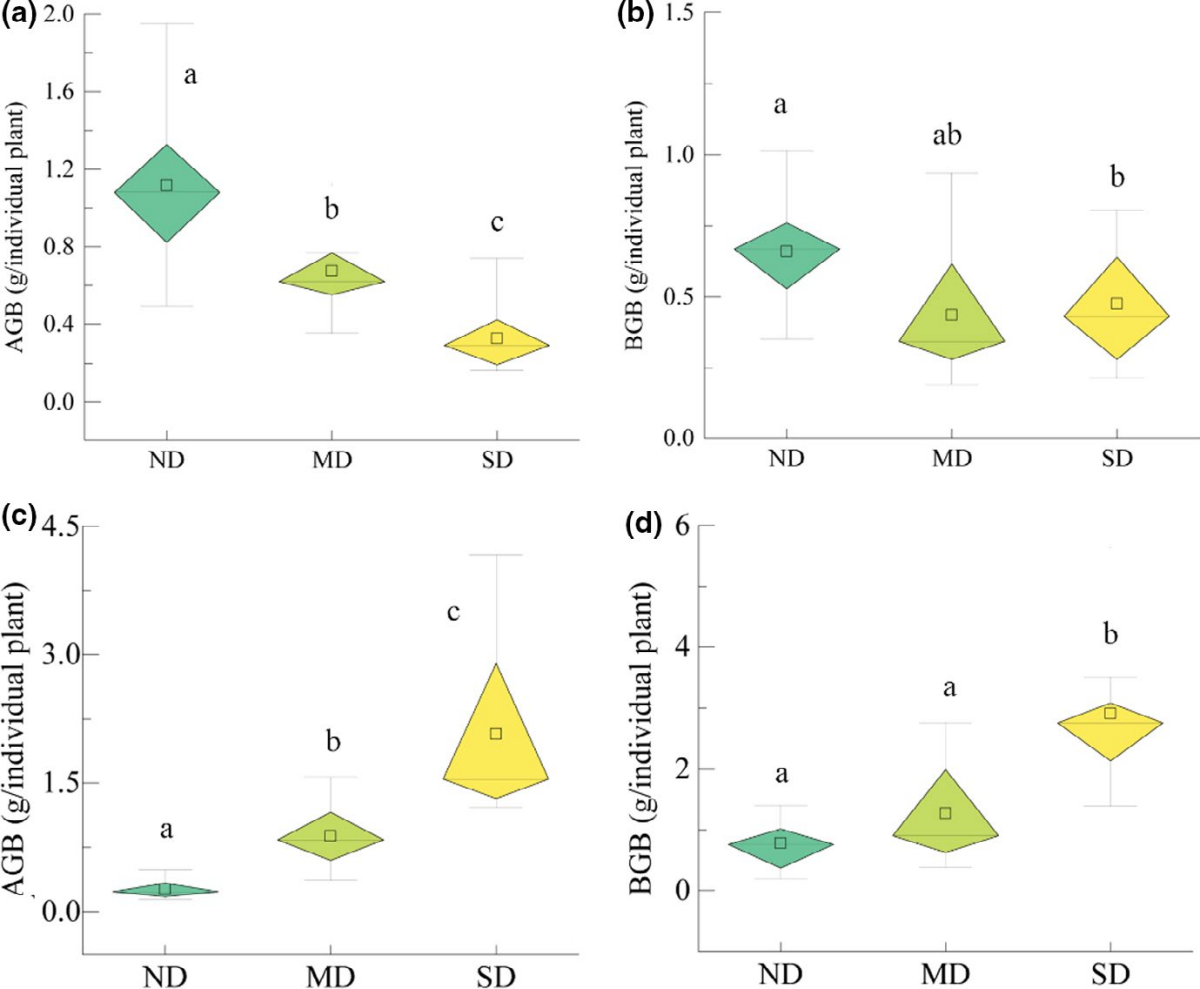
Box and whisker plots showing the aboveground biomass (AGB) in grasses (a) and forbs (c), as well as the belowground biomass (BGB) in grasses (b) and forbs (d) along grassland degradation gradients (including nondegradation [ND], moderate degradation [MD], and severe degradation [SD]). Different letters indicate significant differences between degradation gradients (Tukey's test, *p* < 0.05)

With increasing degeneration, the AGB of grasses exhibited a significant (*p* < 0.05) decrease, with the values of 1.19 g/individual (coefficient of variation, CV = 0.32), 0.69 g/individual (CV = 0.39), and 0.32 g/individual (CV = 0.55) obtained in ND, MD, and SD, respectively (Figure 2a). In contrast, a significant (*p* < 0.05) increase of AGB in forbs was observed, with the values of 0.26 g/individual (CV = 0.41), 0.88 g/individual (CV = 0.42), and 2.07 g/individual (CV = 0.47) in ND, MD, and SD, respectively (Figure 2c). There was no significant difference in BGB in grasses between ND, MD, and SD (Figure 2b). Meanwhile, BGB in forbs showed no significant increase from ND (0.78 g/individual, CV = 0.53) to MD (1.27 g/individual, CV = 61), but a significant (*p* < 0.05) value was obtained in SD (2.91 g/individual, CV = 0.39) (Figure 2d).

### 3.2 The trade‐off between AGB and BGB of grasses and forbs

**FIGURE 3:**
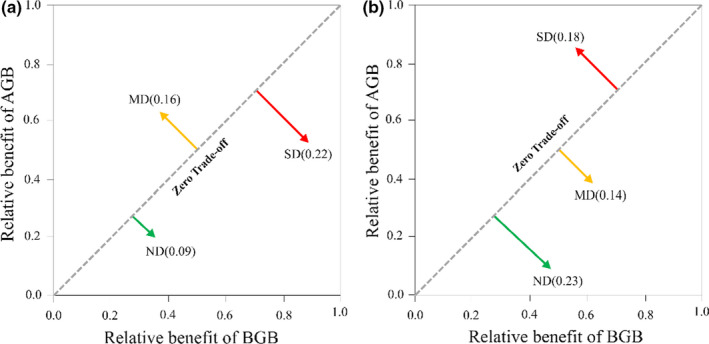
The trade‐off between aboveground biomass (AGB) and belowground biomass (BGB) for grasses (a) and forbs (b) along grassland degradation gradients (including nondegradation [ND], moderate degradation [MD], and severe degradation [SD])

The trade‐off between AGB and BGB of grasses in ND (0.09) was lower than that in MD (0.16) and SD (0.22, Figure 3a). The trade‐off of grasses was shifted from the BGB in ND (lower in 1:1 line) to the AGB in MD (upper in 1:1 line) and toward the BGB in SD (lower in 1:1 line, Figure 3a). For forbs, the trade‐off decreased from 0.23 to 0.14 (lower in 1:1 line) as the degradation increased from ND to MD and then increased to 0.18 (upper in 1:1 line) in SD (Figure 3b).

### 3.3 Linking environmental factors to the trade‐off of grasses and forbs

**FIGURE 4:**
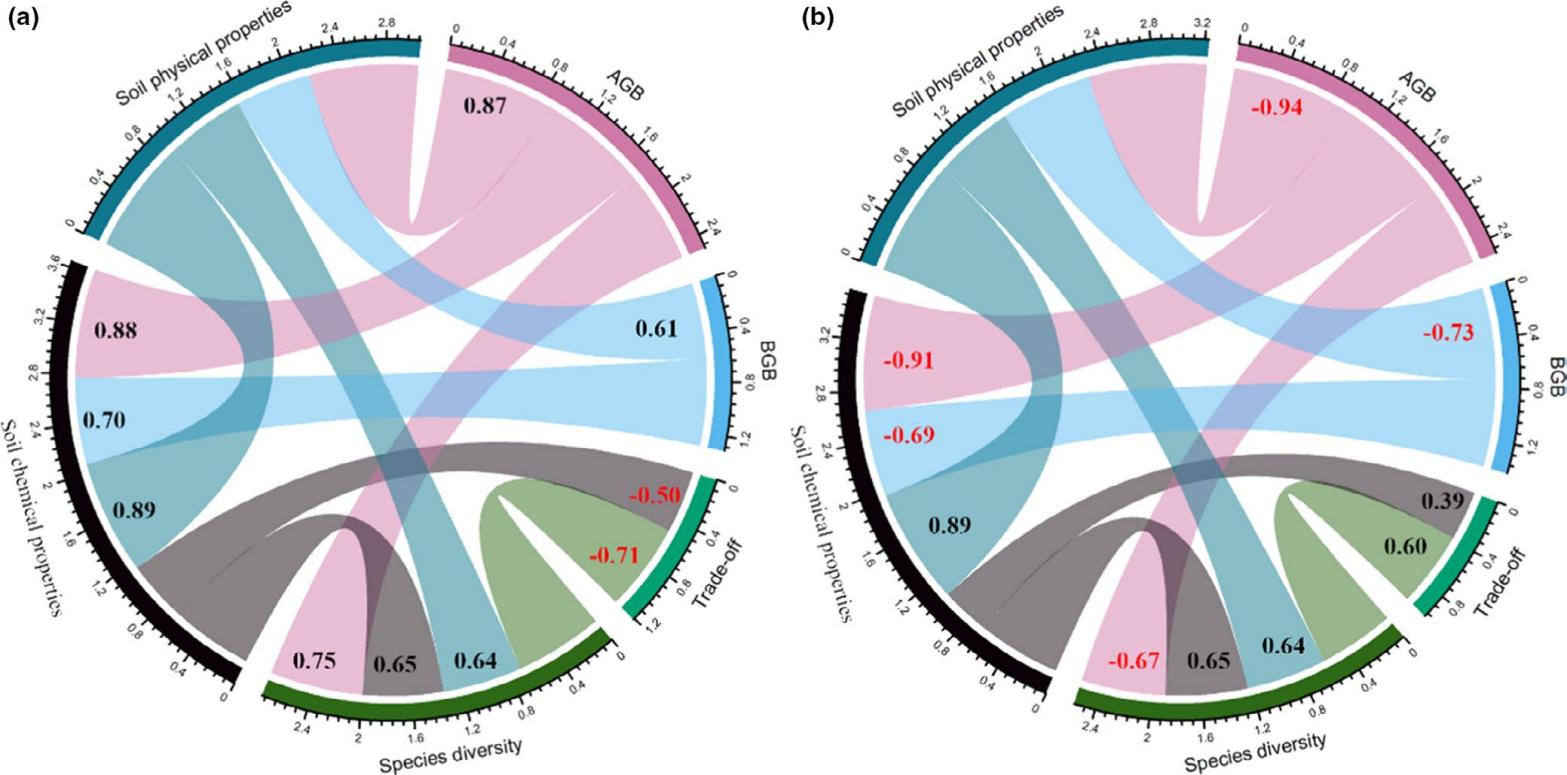
Relationships between the trade‐off and environmental factors for grasses (a) and forbs (b) across grassland degradation gradients. Black and red numbers represent significantly (*p* < 0.05) positive and negative effects, respectively

From ND to SD, Figure 4a shows that the biomass trade‐off in grasses was significantly and negatively correlated with community species diversity (*R* ^2^ = −0.71; *p* < 0.05) and soil chemical properties (*R* ^2^ = −0.50; *p* < 0.05), respectively. Meanwhile, the species diversity in the community was significantly (*p* < 0.05) affected by AGB (*R* ^2^ = 0.75), soil chemical properties (*R* ^2^ = 0.65), and soil physical properties (*R* ^2^ = 0.64, Figure 4a).

In forbs, the primary factors that positively affected the trade‐off were species diversity in the community (*R* ^2^ = 0.60; *p* < 0.05) and soil chemical properties (*R* ^2^ = 39; *p* < 0.05, Figure 4b). Our results also suggested that AGB, BGB, and soil physical properties had significant (*p* < 0.05) effects on the community species diversity and soil chemical properties (Figure 4b). These results demonstrated that there were contrasting relationships between the trade‐offs and factors in grasses and forbs.

### 3.4 Control mechanisms of environmental factors on the trade‐off of grasses and forbs

**FIGURE 5:**
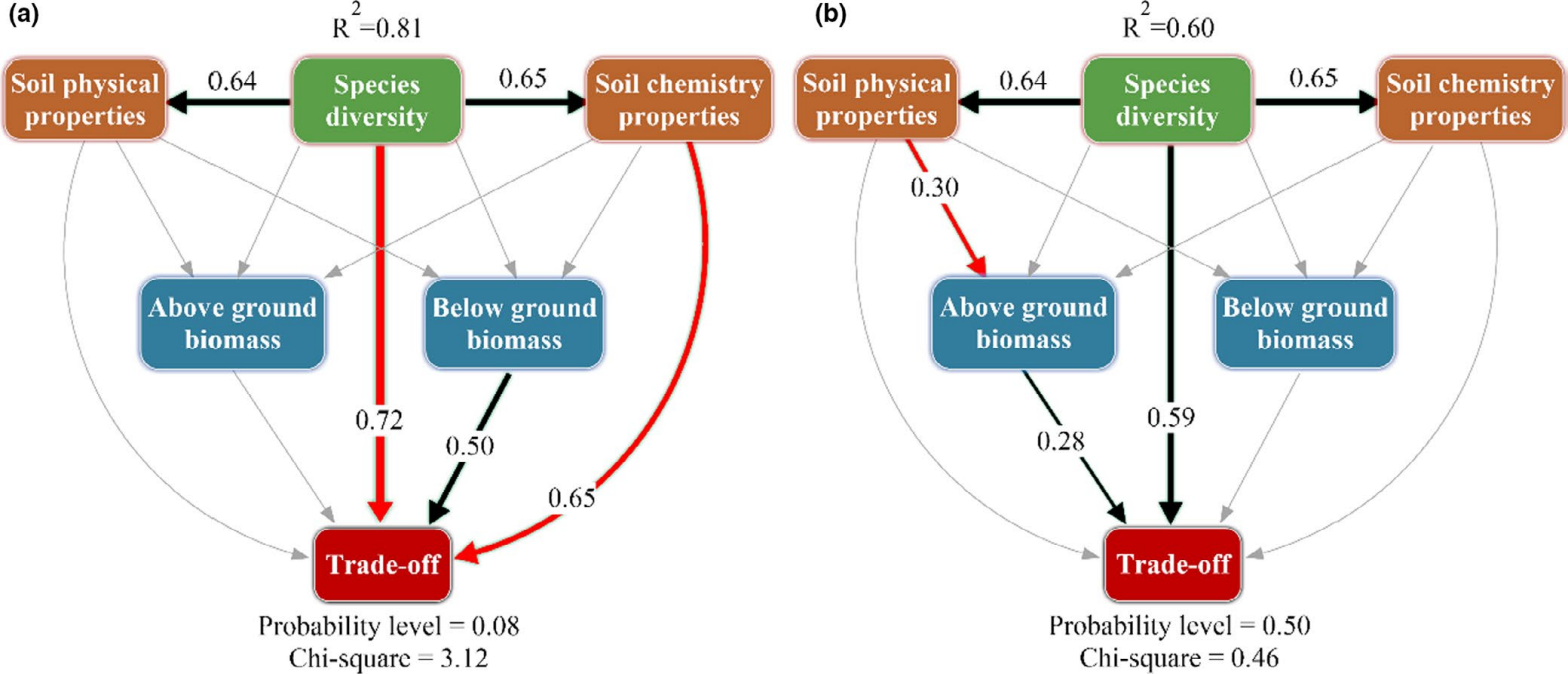
Mechanisms for the trade‐off in grasses (a) and forbs (b) across grassland degradation gradients. Structure equation modeling (SEM) examining the standard total effects of environmental factors on the trade‐off. Black solid lines and solid red lines represent significantly (*p* < 0.05) positive and negative relationships, respectively, while the gray lines indicate insignificant relationships

Path analyses demonstrated that 81% (grasses) and 60% (forbs) of the trade‐off between AGB and BGB were explained by our model (Figure 5). Specifically, community species diversity and soil chemical properties generated significant (*p* < 0.05) negative effects on the trade‐offs in grasses, with the standard total effects of −0.72 and −0.65, respectively (Figure 5a). In contrast, BGB had a significant (*p* < 0.05) positive effect on the biomass trade‐off (scored at 0.50, Figure 5a).

In forbs, the trade‐off between AGB and BGB was significantly and positively affected by species diversity (scored at 0.59) and AGB (scored at 0.28, Figure 5b). And there were indirectly significant effects of soil physical properties on the biomass trade‐off. Taken together, these results demonstrate that the trade‐offs in grasses and forbs can be explained by species diversity through either indirect or direct effects on soil properties and biomass.

## 4 DISCUSSION

### 4.1 Size and direction of biomass partitioning in grasses and forbs

Along degraded grassland gradients, the range of biomass trade‐off values for grasses (0.13) and forbs (0.41) in our study (Figure 3a,b) was larger than the range detected in grazing grasslands (0.06) (Sun, Ma, et al., 2018) or in recovering grasslands (0.04) (Liu, Zhang, Sun, Wang, et al., 2020). This demonstrates that degradation can leave a mark on biomass partitioning. In ND, for grasses, the biomass partitioning trade‐off (0.09, Figure 3a) was comparable with the trade‐off (0.07) obtained in grazing exclusion (Sun, Ma, et al., 2018). In ND, the direction of the trade‐off for grasses was the same as that in alpine meadow (Peng, Xue, You, et al., 2020). In SD, the direction of trade‐off for grasses in this study was different from that in Peng, Xue, Li, et al. (2020), while the direction (above the 1:1 line, Figure 3b) of the trade‐off for forbs was similar to that in Peng, Xue, You, et al. (2020). This is not only because the focus of our study is on the biomass partitioning trade‐off of different plant functions, which would either be the same or different from the trade‐off obtained at the community level; but also because of the shifting of dominant species with grassland degradation (Peng, Xue, You, et al., 2020; Wang et al., 2009).

**FIGURE 6:**
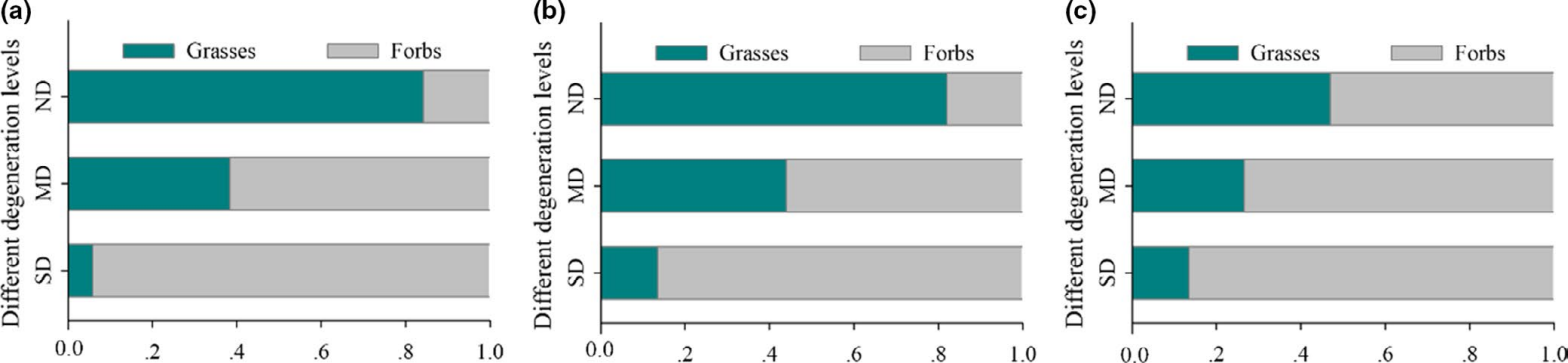
Along grassland degradation gradients (including nondegradation [ND], moderate degradation [MD], and severe degradation [SD]), the percentage coverage of grasses and forbs to total coverage (a), aboveground biomass of grasses and forbs to total aboveground biomass (b), and belowground biomass of grasses and forbs to total belowground biomass (c)

Similar to the results of previous studies, our results suggested that, with an increase in degradation, the trade‐off value of forbs significantly shifted from negative to positive (Zhang et al., 2020; Zhang & Sun, 2020). Specifically, from ND to SD, the increased AGB ratio was higher than that of BGB for forbs in our study (Figure 6b,c), which moved the trade‐off away and above the 1:1 line (Figure 3b). This suggests that forbs were more competitive for light in the aboveground than grasses in degraded grasslands and indicates that there was a retrogressive succession in plant communities (Sun, Schleuss, et al., 2018; Wang et al., 2009).

### 4.2 Mechanism of environmental factors in biomass partitioning

Biomass partitioning strategies reflect the survival, growth, and reproduction of plants in response to environmental variation (Shipley & Meziane, 2002). Across grassland degraded gradients, there were contrasting relationships between the trade‐off and species diversity (soil properties) for grasses and forbs. The belowground resource requirement of forbs might be lower than the grasses (Terrer et al., 2021), this will be supported by the negative correlation of trade‐off between chemical properties for grasses (Figure 4a) but the positive correlation for forbs (Figure 4b). Based on this result, it was reasonable to confirm that there were divergent effects of factors on the biomass trade‐off for grasses and forbs. The magnitude and direction of environmental effects on AGB and BGB depend on several aspects, including community species diversity, soil nutrients, and plant survival strategies.

Across the degraded gradients, our results showed that community species diversity was a more important factor for the trade‐offs in grasses and forbs than soil properties (Figure 5). Biomass allocation in plant communities can be mediated by interspecific heterogeneity that modifies the biomass trade‐offs between AGB and BGB for different plant groups (Gao et al., 2011; Peng, Xue, You, et al., 2020). In the process of degradation, changes in habitats cause the invasion of forbs (Wang et al., 2009; Xu et al., 2015), leading to the decrease of species diversity within the plant community. Grasses gradually lose their ascendancy in the community, while forbs become dominant species due to their strong competitiveness for nutrients (Dormann et al., 2000; Pang et al., 2019). This decrease of community species diversity is mainly because the disturbance of climate or human activities in alpine meadow changes the physical and chemical properties of the soil (Guo et al., 2019; Wang et al., 2014, 2016). The disturbed soil and habitat then provide a niche for invasive species of forbs (Peng, Xue, You, et al., 2020; Zhang et al., 2005). Few species occurs because grassland degradation, soil nutrient reduction (Table 1) aggravates the already limited resource availability for grasses (negative effect of soil nutrients on the trade‐off, Figure 5a), but this is not so for forbs (nonsignificant effect of soil nutrients on the trade‐off Figure 5b). This is because forbs are more adaptive to the resource‐limited environment (Miehe et al., 2011; Wang et al., 2009). Finally, forbs allocated more biomass aboveground (Figure 3b) to compete for resources in lower species diversity regions, and grasses allocated more biomass belowground (Figure 3a) for survival after grassland degradation.

Moreover, there was a close relationship between grassland degradation and soil degradation because the soil is the foundation for alpine meadow (Zhang et al., 2018). As the soil fertility levels decreased significantly (Table 1), a significant decrease in the biomass of grasses and a significant increase in the biomass of forbs occurred (Figure 4). In ND, our results indicated that both surface and deep soil moistures, as well as nutrients, would be used by grasses with the aid of their long roots (Figure S4a) and dominant BGB (Figure 6a) within the community. In contrast, forbs can only utilize resources from the surface layer due to their short roots (Figure S4b), as well as their low ratio of BGB (Figure 6b). After grassland degradation, grasses allocated more biomass belowground (Figure 3a), whereas forbs allocated more biomass aboveground (Figure 3b). Because forbs increase coverage and leaf area to enhance the photosynthetic uptake of carbon and AGB (Liu, Zhang, Sun, Li, et al., 2020; Peng, Xue, You, et al., 2020), the trade‐off moved away and stayed above the 1:1 line (Figure 3b). Because of their low coverage (low competitive ability for light) (Figure 6a), grasses struggle for survival in degraded environments (Wang et al., 2009; Zhang et al., 2020), leading to the trade‐off below the 1:1 line (Figure 3a). These divergent biomass partitioning patterns indicated that soil traits were also important driving forces in grassland community succession (Zhang et al., 2018) and biomass partitioning (Roa‐Fuentes et al., 2012; Sun, Ma, et al., 2018).

Generally, plants with different functions do not consistently respond to grassland degradation, they adjust their biomass allocation strategies according to the environment (Poorter et al., 2011; Shipley & Meziane, 2002; Weiner & Systematics, 2004). Our findings suggested that the plant community had evolved as two contrasting strategies for biomass allocation in degraded grasslands. For grasses, in both ND and SD, the trade‐offs below the 1:1 line (Figure 3a) indicated a “conservative” strategy in which plants with belowground trade‐offs depend on gigantic roots for soil resources. For forbs, whether the big trade‐off was below the 1:1 line in ND or the big trade‐off was above the 1:1 line in SD (Figure 3b), both demonstrated that forbs had more flexible survival strategies than grasses in response to degradation (Dormann et al., 2000; Pang et al., 2019). The “opportunistic” strategies in forbs, in which plants with high variation biomass trade‐off, were enabled to allocate photosynthetic carbon more optimally (Ma et al., 2018; Roumet et al., 2016).

In summary, we concluded that the degradation of alpine meadow could generate significantly different effects on plant biomass allocation for grasses and forbs. Specifically, for grasses, plant biomass allocation trade‐offs were directed mainly belowground. Meanwhile, for forbs, the plant biomass allocation trade‐off shifted from belowground in ND to aboveground in SD. Moreover, our results demonstrated that the biomass trade‐offs in grasses and forbs could be explained by community species diversity through either direct or indirect effects on soil properties and biomass. Thus, plant communities might have evolved as two contrasting strategies of biomass allocation patterns (a “conservative” strategy in grasses and an “opportunistic” strategy in forbs) in response to degradation. In terms of grassland degradation, our work has expanded the study of biomass allocation within the community to individual plants. The knowledge of degradation mechanisms obtained in this study will advance our knowledge of survival strategies for different plant functional groups.

## CONFLICT OF INTEREST

The authors declare no competing interests.

## AUTHOR CONTRIBUTIONS

**Tiancai Zhou:** Data curation (equal); Methodology (equal); Software (equal); Writing‐original draft (equal); Writing‐review and editing (equal). **Jian Sun:** Methodology (equal); Writing‐review and editing (equal). **Ning Zong:** Data curation (equal); Funding acquisition (equal); Writing‐review and editing (equal). **Ge Hou:** Data curation (equal); Writing‐review and editing (equal). **Peili Shi:** Conceptualization (equal); Funding acquisition (equal); Resources (equal); Writing‐review and editing (equal).

## Supporting information

Figures S1‐S4

## Data Availability

Data will be submitted to the Dryad Digital Repository: https://doi.org/10.5061/dryad.0gb5mkm1v.
