# Supplementary material for: Community species diversity mediates the trade‐off between aboveground and belowground biomass for grasses and forbs in degraded alpine meadow, Tibetan Plateau
Source: Ecol Evol. 2021 Aug 29;11(19):13259–67. doi: 10.1002/ece3.8048 (PMC8495812; doi:10.1002/ece3.8048)
Supplement: Supplementary file 1 — Figures S1‐S4 [file ECE3-11-13259-s001.docx]

**Supporting information**

Community species diversity mediates the trade-off between aboveground and belowground biomass for grasses and forbs in degraded alpine meadow, Tibetan Plateau

**Supplement Figures:**


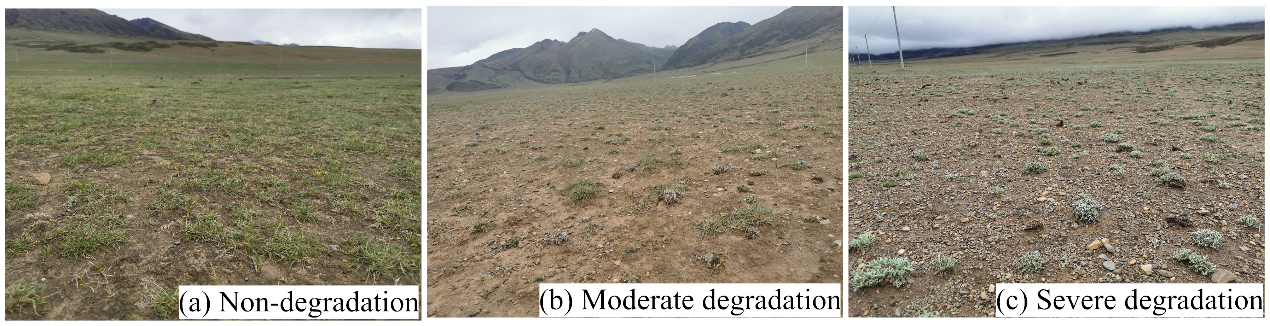


**Supplement Figure 1** The landscape in non-degraded (dominate species of *Stipa capillacea*, a), moderately degraded (dominate species of *Stipa capillacea* and *Anaphalis xylorhiza*, b) and severely degraded (dominate species of *Anaphalis xylorhiza*, c) grasslands.


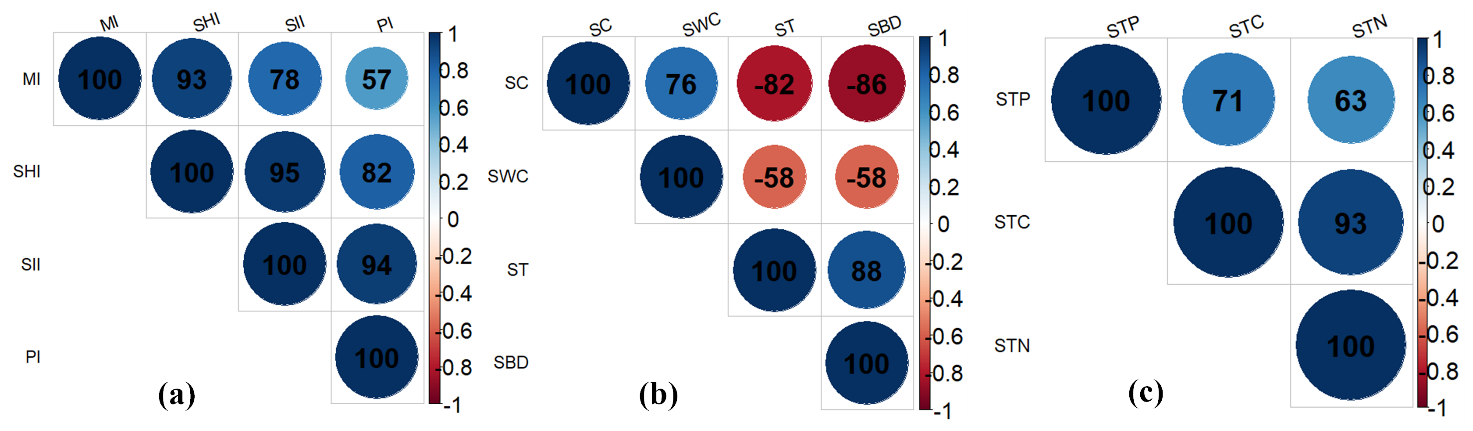


**Supplement Figure 2** Heat map showing correlations among all examined variables: species diversity indexes of *Shannon-Wiener Index* (SHI), *Simpson Index* (SI), *Margalef Index* (MI)and *Pielou Index* (PI) in graph a; soil physical properties of soil compactness (SC), water content (SWC), temperature (ST), and bulk density (SBD) in graph b; and soil chemical properties of soil total carbon (STC), soil total nitrogen (STN) and soil total phosphorus (STP) in graph c.


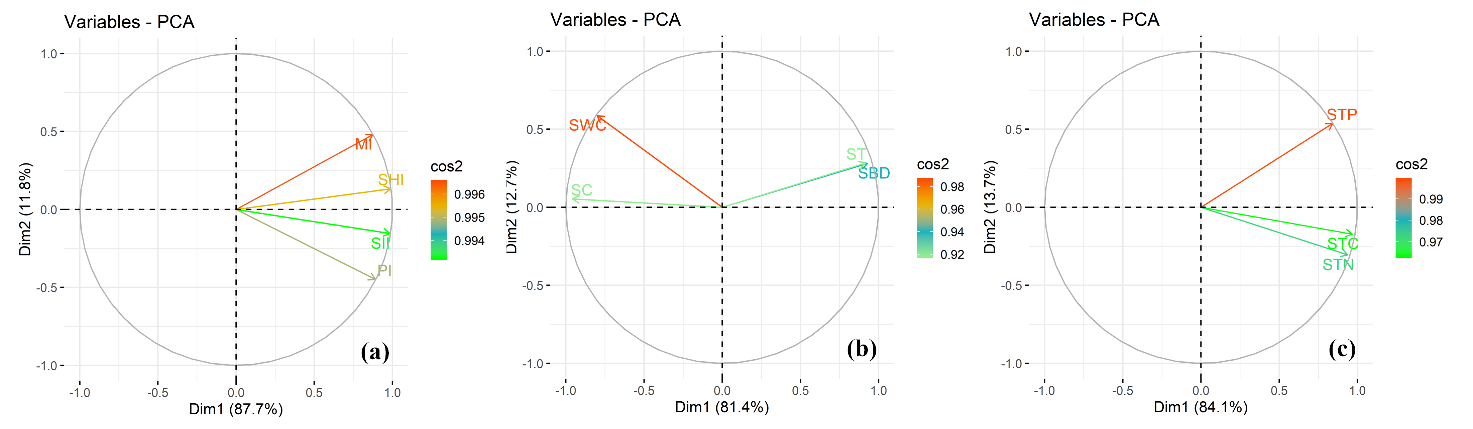


**Supplement Figure 3** Principal components analysis (PCA) of the examined variables: species diversity indexes in graph a; soil physical properties in graph b; and soil chemical properties in graph c. Please see abbreviations for variables in Supplement Figure 3.


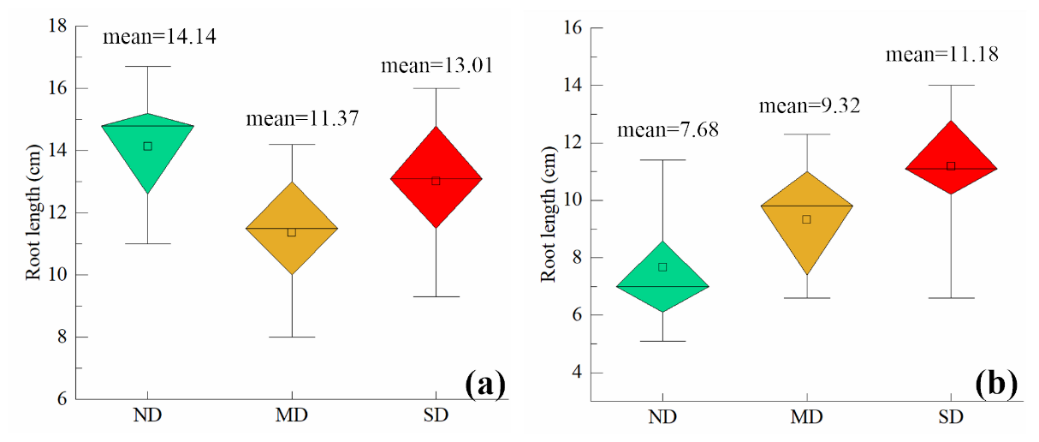


**Supplement Figure 4** Box-and-whisker plots show the root length in grasses (a) and forbs (b) along grassland degradation gradients (including non-degradation [ND], moderate degradation [MD] and severely degradation [SD]).
